# Emergency Cesarean Delivery Complicated by Massive Postpartum Hemorrhage Revealing an Undiagnosed Unicornuate Uterus: A Case Report

**DOI:** 10.7759/cureus.108689

**Published:** 2026-05-11

**Authors:** Soumil Sharma, Soheil Farnaghi

**Affiliations:** 1 Obstetrics and Gynaecology, Logan Hospital, Brisbane, AUS

**Keywords:** caesarean section, gestational hypertension, müllerian anomaly, nuchal translucency, postpartum haemorrhage, prenatal diagnosis, unicornuate uterus, urinary tract anomaly

## Abstract

Increased first-trimester nuchal translucency (NT) may be associated with chromosomal, genetic, and structural abnormalities, although some pregnancies progress with normal invasive testing and reassuring interval imaging. This case involved a 32-year-old primigravida referred at 12+5 weeks’ gestation following the identification of markedly increased NT measuring 5.6 mm, with associated body wall oedema. Chorionic villus sampling demonstrated normal fluorescence in situ hybridisation (FISH) and chromosomal microarray results, while subsequent antenatal imaging showed resolution of the body wall oedema without identifiable fetal structural anomalies. Later in pregnancy, gestational hypertension developed, requiring antihypertensive therapy and induction of labour at 38 weeks. Following failure to progress and an abnormal cardiotocograph, an emergency caesarean section was performed. Intraoperatively, a previously undiagnosed unicornuate uterus with an absent left fallopian tube, ovary, uterine artery and ureter was identified. Delivery was complicated by uterine angular extension, severe uterine atony, persistent haemorrhage and difficult pelvic anatomy, progressing to massive postpartum haemorrhage with an estimated cumulative blood loss of 10 L. Despite escalation through uterotonics, compression sutures, vascular ligation, vaginal packing and Bakri balloon tamponade, definitive haemostasis required hysterectomy, excision of the cervical remnant, right internal iliac ligation, and multidisciplinary surgical input. The patient required massive transfusion and intensive care admission but recovered postoperatively. This case highlights the complexity of counselling pregnancies with isolated increased NT despite normal genetic testing and illustrates how previously occult Müllerian and urinary tract anomalies may only become evident during obstetric emergency surgery, profoundly influencing haemorrhage control and operative risk.

## Introduction

Increased nuchal translucency (NT) in the first trimester is a well-recognised marker of fetal chromosomal, genetic and structural abnormality. The risk of an adverse outcome increases with NT; for measurements of 5.5 mm or greater, the likelihood of an unaffected live birth is reported to be approximately 30%, even when the fetal karyotype is normal [1,2]. Accordingly, even where invasive genetic testing is reassuring, ongoing surveillance is recommended because increased NT may still be associated with congenital heart disease, hydrops, pregnancy loss and adverse perinatal outcomes [1,2]. Chromosomal microarray offers additional diagnostic yield in fetuses with increased NT and a normal karyotype. At the same time, further single-gene testing may identify residual causes, particularly RASopathies such as Noonan syndrome, in selected cases following a normal microarray [3,4].

Müllerian anomalies are clinically important in obstetrics because they are associated with miscarriage, malpresentation, fetal growth concerns, preterm birth and operative delivery [5-7]. Renal and urinary tract anomalies are a clinically important association, occurring in up to 40% of affected patients, because of the shared embryological development of the genital and urinary systems [5,6]. In contrast, Mayer-Rokitansky-Küster-Hauser (MRKH) syndrome is characterised by uterovaginal aplasia or severe hypoplasia rather than a functional malformed uterus, and associated renal, auditory and cardiac anomalies are more typical of MRKH type 2 or MURCS (Müllerian duct aplasia, renal aplasia, and cervicothoracic somite dysplasia) association [8]. This distinction is relevant in the present case, where natural conception and carriage of an intrauterine pregnancy to term argue against classic MRKH [8].

## Case presentation

A 32-year-old primigravida was referred to maternal fetal medicine after an external ultrasound at 10+6 weeks demonstrated increased NT. Formal assessment at 12+5 weeks showed markedly increased NT measuring 5.6 mm with associated body wall oedema, but no other structural abnormality was identifiable at that gestation. She was counselled regarding the possibility of a normal outcome as well as chromosomal, genetic and structural pathology. Transabdominal chorionic villus sampling was performed without complication. Fluorescence in situ hybridisation (FISH) testing for common aneuploidies was normal, and chromosomal microarray demonstrated no clinically significant copy number abnormality. She later underwent genetic counselling, during which residual single-gene causes were discussed; genomic sequencing was considered, but she was not eligible for the available programme based on the findings at that time. Maternal G-banded karyotyping was also performed because of a family history of trisomy 21 in two cousins, and was normal.

Her history was notable for repair of a congenital cardiac defect in infancy, described as a ventricular septal defect, and mild-to-moderate bilateral hearing impairment requiring hearing aids. Serial structural surveillance was undertaken through maternal fetal medicine. At 17+4 weeks, the nuchal fold measured 3.7 mm, the body wall oedema had resolved, and no structural abnormality was identified. At 21+2 weeks, the nuchal fold remained increased at 5.6 mm, but detailed assessment again demonstrated no structural anomaly. Subsequent third-trimester surveillance remained reassuring, with estimated fetal weight between the 15th and 17th centiles and normal amniotic fluid volume and Doppler studies. Antenatal imaging did not report uterine or renal tract abnormalities.

Additional antenatal issues included RhD-negative status with a RhD-positive fetal phenotype on non-invasive prenatal assessment, iron deficiency treated with oral supplementation, positive anti-thyroid peroxidase antibodies with normal thyroid function and gestational hypertension from 36+3 weeks. She underwent several assessments for elevated blood pressure, with repeated normal pre-eclampsia investigations including platelet count, renal function, liver enzymes and urine protein-creatinine ratio. Labetalol was commenced and progressively increased because of labile blood pressures. Given persistent gestational hypertension, induction of labour was recommended and commenced at 37+6 weeks, with delivery planned at 38 weeks.

Induction commenced with attempted cervical ripening balloon insertion, which was unsuccessful because the catheter could not be passed through the cervix, followed by prostaglandin administration. She subsequently had spontaneous rupture of membranes and required oxytocin augmentation. Labour progress remained poor: after prolonged labour, cervical dilatation persisted at 5 cm despite augmentation, with clinical concern for malposition or asynclitism and significant caput. The cardiotocograph evolved from initially reassuring to abnormal, with complicated and persistent variable decelerations, periods of reduced variability, and later reduced variability with variable decelerations despite adjustment and cessation of oxytocin. In the setting of failure to progress with fetal concern, a category 2 emergency lower-segment caesarean section was undertaken.

A live female infant was delivered in cephalic presentation at 38+0 weeks, weighing 3,090 g. She was born in poor condition with Apgar scores of 2, 4 and 9 at 1, 5 and 10 minutes, respectively, requiring intermittent positive-pressure ventilation, cardiopulmonary resuscitation from 2 to 6 minutes of life, and continuous positive airway pressure until 18 minutes before stabilising. She was admitted to the neonatal intensive care unit for observation and antibiotics following unexpected resuscitation; postnatal examination also identified bilateral talipes, more marked on the right, and she later remained clinically stable. 

At caesarean section, unexpected abnormal pelvic anatomy was encountered, including a unicornuate uterus, absent left fallopian tube and ovary, absent left uterine artery and absence of the left ureteric orifice on cystoscopy. A right-sided uterine angular extension extended towards the uterine artery. Following delivery at 14:41, severe uterine atony and haemorrhage developed, with the caesarean section and initial postpartum haemorrhage management completed by 15:15. The uterine atony was likely multifactorial. While the unicornuate uterus may have contributed through abnormal myometrial architecture and distorted pelvic anatomy, prolonged induction, extended labour with oxytocin augmentation and obstructed labour were additional recognised risk factors for poor uterine tone in this case. Management was escalated sequentially with carbetocin, repeated carboprost, ergometrine, tranexamic acid, B-Lynch compression suture, haemostatic suturing of the posterior uterine venous plexus, uterine artery ligation, vaginal packing and Bakri balloon tamponade. Ongoing bleeding prompted activation of the massive transfusion protocol at 16:40, and, despite these measures, the decision for hysterectomy was made at 17:25. Persistent haemorrhage from the cervical remnant in the setting of distorted anatomy necessitated re-opening, completion excision of the remnant cervix, right ureterolysis and right internal iliac artery ligation with senior multidisciplinary surgical input; the procedure concluded at 22:59. Total cumulative estimated blood loss was approximately 10 L, and the patient received 18 units of packed red blood cells in addition to fresh frozen plasma, cryoprecipitate and albumin.

**Figure 1:**
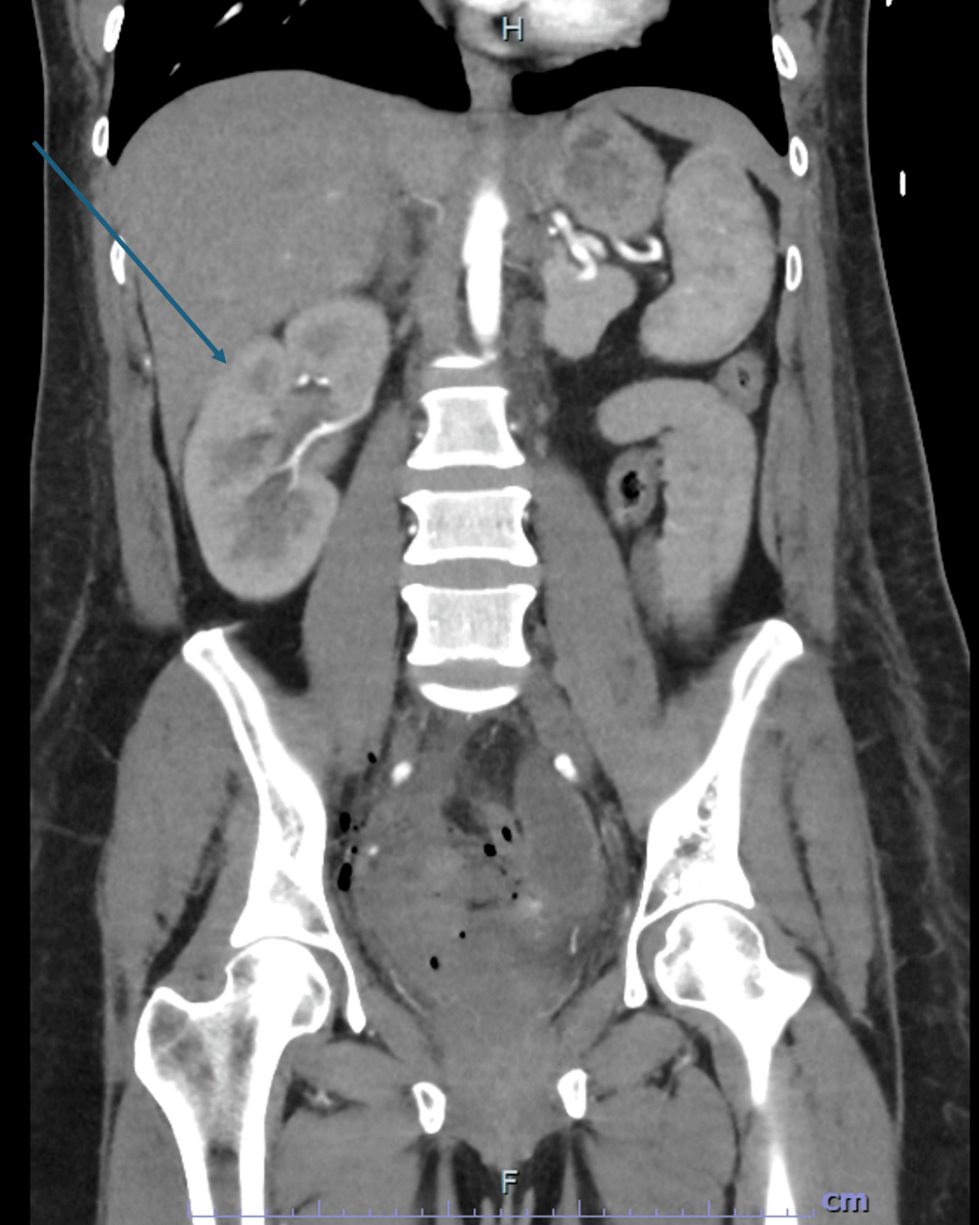
Coronal CT intravenous pyelogram demonstrating congenital absence of the left kidney and left renal artery, with compensatory hypertrophy of the right kidney (blue arrow). This confirmed the postoperative diagnosis of an associated renal tract anomaly and supported that the absent left ureteric orifice identified intraoperatively reflected congenital renal agenesis rather than iatrogenic ureteric injury.

**Figure 2:**
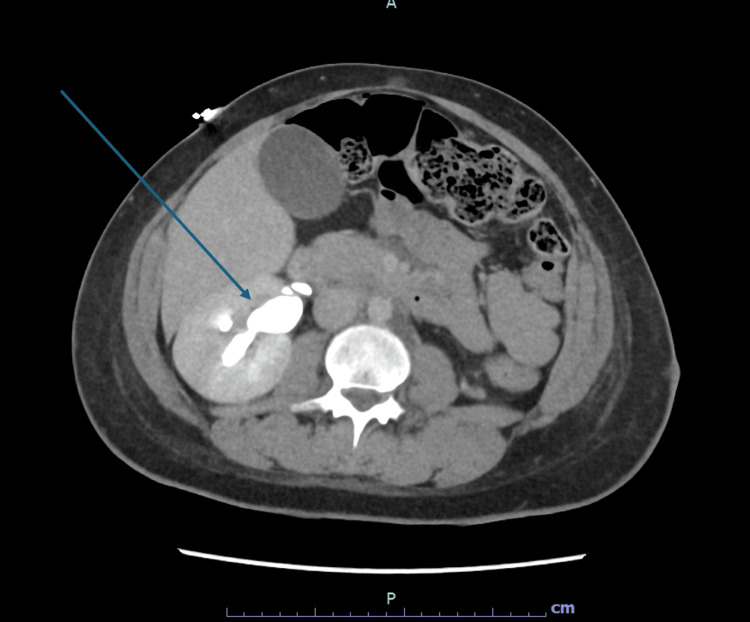
Axial CT intravenous pyelogram demonstrating a hypertrophied right kidney with a tortuous contrast-opacified right ureter coursing around the renal outline (blue arrow). This highlights the complex anatomy of the patient’s solitary functioning urinary tract, relevant to the need for careful right ureteric identification and ureterolysis during haemorrhage control.

**Figure 3:**
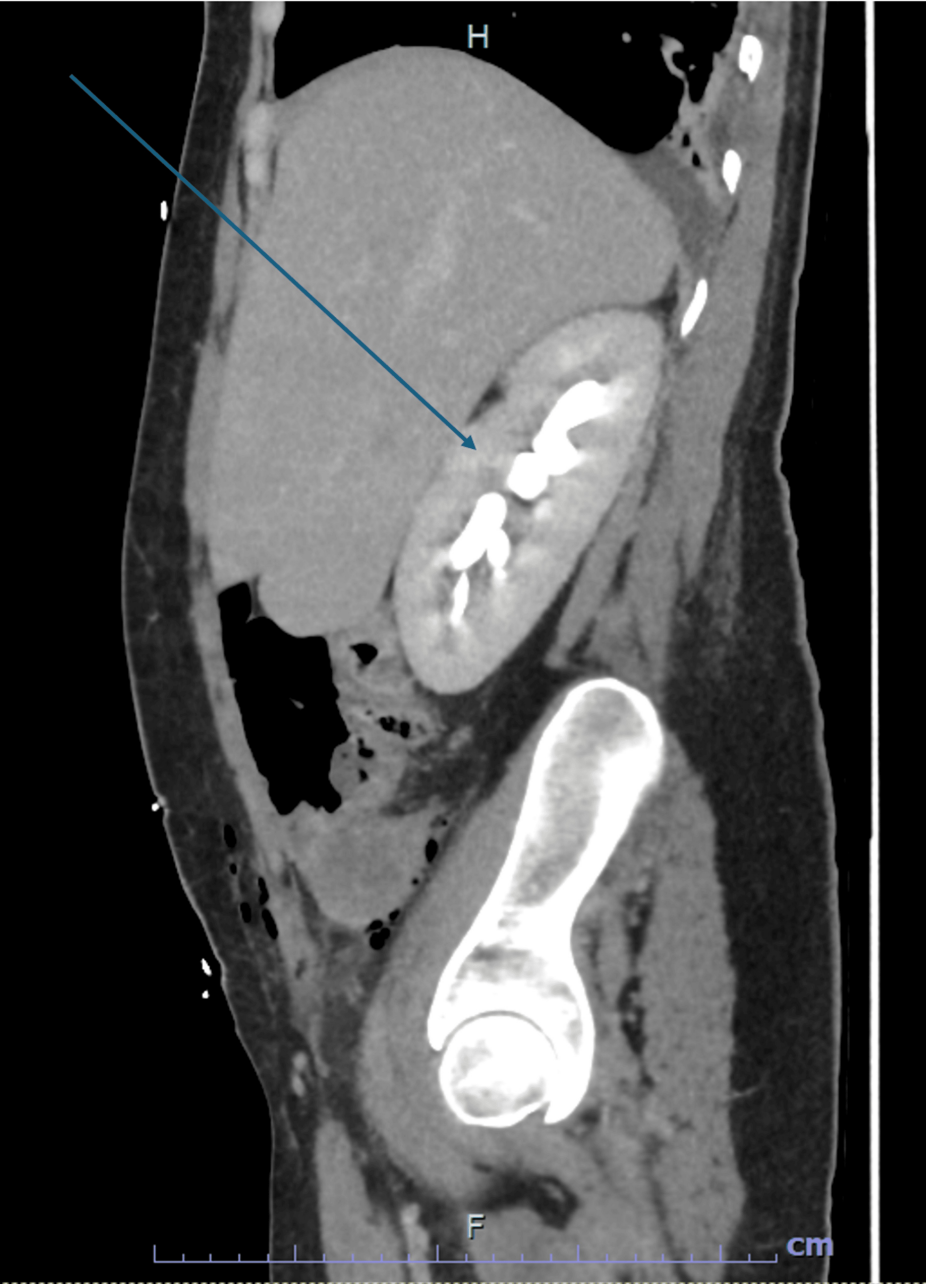
Sagittal CT intravenous pyelogram demonstrating compensatory hypertrophy of the right kidney with mild right hydronephrosis (blue arrow). This further illustrates the altered anatomy of the solitary right renal system, emphasising the operative importance of preserving the only functioning ureter during emergency pelvic surgery.

Postoperatively, she was admitted to intensive care, where serial postoperative blood results demonstrated profound acute anaemia and thrombocytopenia following massive haemorrhage and transfusion, reflecting the physiological burden of severe obstetric bleeding and resuscitation (Table 1). She remained intubated initially, was extubated the following day, and recovered with multidisciplinary input from intensive care, obstetrics, haematology, infectious diseases and urology. Postoperative CT intravenous pyelography confirmed congenital absence of the left kidney and left renal artery, with compensatory hypertrophy of the solitary right kidney, supporting the intraoperative impression that the absent left ureteric orifice represented a congenital Müllerian-renal anomaly rather than iatrogenic ureteric injury (Figures 1-2). Mild right hydronephrosis and a tortuous right ureter with multifocal narrowing were also demonstrated, underscoring the importance of ureteric identification and preservation during haemorrhage control in distorted pelvic anatomy (Figures 2-3). She made a progressive postoperative recovery and was discharged after a period of ward monitoring.

**Table 1:** Preoperative and postoperative laboratory results. MCV, mean corpuscular volume; MCH, mean corpuscular haemoglobin

Test	Reference range	Pre-op bloods	Post-op bloods
Haemoglobin	~115-165 g/L	119	63
White cell count	~4.0-11.0 × 10⁹/L	20.5	9.5
Platelet count	~150-400 × 10⁹/L	300	56
Haematocrit	~0.36-0.47 L/L	0.36	0.18
MCH	~27-32 pg	29.9	29.4
Red cell count	~3.8-5.8 × 10¹²/L	3.98	2.14
MCV	~80-100 fL	90	86
Neutrophils	~2.0-7.5 × 10⁹/L	18.06	7.4
Lymphocytes	~1.5-4.0 × 10⁹/L	1.31	1.23
Monocytes	~0.2-0.8 × 10⁹/L	1.06	0.82
Eosinophils	~0.04-0.4 × 10⁹/L	0.00	0.00
Basophils	~0.00-0.10 × 10⁹/L	0.02	0.02

As she was RhD-negative and had been exposed to RhD-positive blood products during massive transfusion, Haematology and Transfusion Medicine were consulted regarding postpartum RhIg. In view of the hysterectomy, RhIg was not administered, as the limited benefit for future pregnancy prevention was judged to be outweighed by the acute risk of haemolysis; follow-up antibody screening was arranged.

## Discussion

This case is notable for two intersecting themes: the antenatal challenge of counselling and surveillance for markedly increased NT with normal invasive genetic testing, and the operative consequences of an unrecognised Müllerian anomaly with associated urinary tract abnormalities in an obstetric emergency.

The first-trimester finding of NT 5.6 mm with body wall oedema placed the pregnancy in a high-risk category despite subsequent normal FISH and chromosomal microarray results. Increased NT is a non-specific marker that remains associated with structural malformations and genetic syndromes even when first-line cytogenetic testing is normal [1-4]. The reassuring interval scans in this case illustrate the residual uncertainty that persists after normal invasive testing and the rationale for continued surveillance [1,2].

The second striking feature was the intraoperative diagnosis of a unicornuate uterus with absent left adnexa and left-sided urinary tract structures. Unicornuate uterus is a recognised Müllerian anomaly associated with renal tract anomalies and adverse reproductive outcomes, including malpresentation, preterm birth, caesarean delivery and reduced live birth rates [5-7]. Distorted vascular and pelvic anatomy likely contributed to the difficulty of haemorrhage control in this case. The combination of uterine angular extension, atony, abnormal uterine vasculature, absent left uterine artery and absent left ureter significantly complicated surgical decision-making. Progressive haemorrhage despite stepwise conservative and surgical measures culminated in hysterectomy, later completion cervical excision and internal iliac ligation [5-7].

This case also reinforces the value of multidisciplinary escalation during catastrophic obstetric haemorrhage. Contemporary reviews of postpartum haemorrhage management emphasise rapid escalation from uterotonics to tamponade, compression sutures, devascularisation procedures, transfusion support and hysterectomy where conservative measures fail [9,10]. The present case followed that pattern, but the altered congenital anatomy made definitive control substantially more difficult. Survival following a 10 L haemorrhage and massive transfusion underscores the importance of structured haemorrhage protocols and access to subspecialty support [9,10].

The question of MRKH type 2 was also relevant in the interpretation of the congenital findings. However, MRKH is defined primarily by congenital absence or severe hypoplasia of the uterus and upper vagina rather than the presence of a functional unicornuate uterus capable of sustaining pregnancy [8]. Although this patient had associated renal, auditory and congenital cardiac abnormalities, the overall phenotype is more consistent with a complex Müllerian anomaly with associated urinary tract and adnexal maldevelopment than with classic MRKH type 2 [5,6,8].

## Conclusions

This case describes a primigravid pregnancy complicated by markedly increased first-trimester NT and body wall oedema with normal invasive prenatal genetic testing, followed by gestational hypertension, emergency caesarean delivery, massive postpartum haemorrhage and life-saving hysterectomy. A previously undiagnosed unicornuate uterus with absent left adnexa and urinary tract structures was identified only intraoperatively, and substantially complicated haemorrhage control. The case highlights the ongoing uncertainty attached to increased NT despite normal genetic testing and the importance of anticipating altered pelvic anatomy, multidisciplinary escalation and postoperative counselling when catastrophic haemorrhage reveals occult congenital anomalies.
